# Pericytes modulate islet immune cells and insulin secretion through Interleukin-33 production in mice

**DOI:** 10.3389/fendo.2023.1142988

**Published:** 2023-03-09

**Authors:** Guzel Burganova, Anat Schonblum, Lina Sakhneny, Alona Epshtein, Tomer Wald, Mika Tzaig, Limor Landsman

**Affiliations:** Department of Cell and Developmental Biology, Sackler Faculty of Medicine, Tel Aviv University, Tel Aviv, Israel

**Keywords:** beta-cell activity, islet vasculature, islets of Langerhans, pancreatic pericytes, Interleukin-33

## Abstract

**Introduction:**

Immune cells were recently shown to support β-cells and insulin secretion. However, little is known about how islet immune cells are regulated to maintain glucose homeostasis. Administration of various cytokines, including Interleukin-33 (IL-33), was shown to influence β-cell function. However, the role of endogenous, locally produced IL-33 in pancreatic function remains unknown. Here, we show that IL-33, produced by pancreatic pericytes, is required for glucose homeostasis.

**Methods:**

To characterize pancreatic IL-33 production, we employed gene expression, flow cytometry, and immunofluorescence analyses. To define the role of this cytokine, we employed transgenic mouse systems to delete the *Il33* gene selectively in pancreatic pericytes, in combination with the administration of recombinant IL-33. Glucose response was measured *in vivo* and *in vitro*, and morphometric and molecular analyses were used to measure β-cell mass and gene expression. Immune cells were analyzed by flow cytometry.

**Resuts:**

Our results show that pericytes are the primary source of IL-33 in the pancreas. Mice lacking pericytic IL-33 were glucose intolerant due to impaired insulin secretion. Selective loss of pericytic IL-33 was further associated with reduced T and dendritic cell numbers in the islets and lower retinoic acid production by islet macrophages.

**Discussion:**

Our study demonstrates the importance of local, pericytic IL-33 production for glucose regulation. Additionally, it proposes that pericytes regulate islet immune cells to support β-cell function in an IL-33-dependent manner. Our study reveals an intricate cellular network within the islet niche.

## 1 Introduction

Insulin secretion is a complex and tightly regulated process that is dependent on both systemic and local signals. β-Cells respond to a variety of inputs from the islet microenvironment. The islet microenvironment comprises various cell populations, such as neuronal, immune, and vascular cells, which play a crucial role in guiding the development, replication, maturation, and function of β-cells (1–4). The activity of these cell populations needs to coordinate effectively to promote glucose regulation through insulin secretion. However, the molecular and cellular interactions between the diverse cell types within the islet microenvironment and how they orchestrate to ensure insulin secretion are still not fully understood.

While islet inflammation can contribute to diabetes, it can also play a beneficial role in glucose homeostasis by supporting β-cell mass and function (1, 5–15). Islets of healthy humans and mice contain immune cells, primarily macrophages, but also T, B, dendritic (DCs), and type 2 innate lymphoid (ILC2s) cells (7, 16). These cells form a tightly-regulated network that supports glucose regulation. Macrophages and DCs were shown to support β-cell function and mass (1, 8, 13, 17, 18), but the role of islet lymphocytes in glucose regulation is currently unclear (16, 19). Additionally, how islet inflammation is regulated to support glucose homeostasis is largely unknown.

IL-33 is a member of the interleukin-1 (IL-1) family of cytokines primarily localized in the nucleus (20, 21). Studies have shown that the localization of IL-33 in the nucleus does not affect gene transcription and that the primary function of its binding to chromatin is to regulate its own extracellular release (22–24). Thus, the biological activity of IL-33 depends on its secretion and subsequent binding to the ST2 receptor (IL-1RL1), which is mainly expressed by immune cells (21, 25). This cytokine has been dubbed “Alarmin” due to its role in activating the immune response; however, recent research has also implicated IL-33 in tissue homeostasis (26), indicating that it plays a complex role in both physiological and pathological conditions such as inflammation, tissue repair, and homeostasis.

The administration of recombinant IL-33 (rIL-33) has been shown to improve mice glucose response by affecting both adipose tissue and the pancreas (7, 27). In agreement, IL-33 null mice display glucose intolerance upon obesity (7). Additionally, rIL-33 administration has been shown to attenuate insulitis in mouse models of type 1 diabetes (28). A recent study by Dalmas et al. demonstrates that in the islets, the expression of ST2 is restricted to ILC2s, which, in response to IL-33 administration, recruit and activate macrophages and DCs (7). Subsequently, macrophages and DCs promote insulin secretion by producing retinoic acid (RA), which has various effects on β-cell function, including the induction of glucose-stimulated insulin secretion, insulin production, and glucokinase activity (7, 29). Thus, rIL-33 activates a cellular network to promote insulin secretion. Although suggested to be cells of mesenchymal origin, the pancreatic source of IL-33 is currently unknown (7). Further, the role of locally produced IL-33 in insulin secretion and glucose homeostasis remains to be elucidated.

Pericytes are contractile mural cells that wrap around capillaries and microvessels in various tissues and organs. Depending on the tissue, these cells have different origins and gene expression patterns (30, 31). In the islets, pericytes have been shown to play an essential role in regulating β-cell function and mass (2, 4, 32–38). Further, this activity can be mediated by secreted factors, including NGF, BMP4, and ECM components, independently of blood flow regulation (32, 34–37). Studies suggest that pericytes may have an immunoregulatory role in various tissues, including the brain and kidneys, by secreting cytokines to control immune cell infiltration and activation (39–44). However, whether pericytes in the islets also have this function has yet to be determined.

Here, we provide evidence for the immunoregulatory role of pancreatic pericytes. First, we identified pericytes as the predominant source of IL-33 in the pancreas. We used transgenic mouse models to selectively delete pericytic IL-33 expression and found that loss of this cytokine caused glucose intolerance due to impaired insulin secretion. Further analyses revealed that pericytic IL-33 regulates the number of immune cells in the islets and their production of RA, indicating that pericytic IL-33 plays an essential role in maintaining glucose homeostasis by regulating immune cells in the islets. Our study indicates that pancreatic pericytes are immunomodulators, revealing an additional regulatory layer of islet inflammation and insulin secretion.

## 2 Materials and methods

### 2.1 Mice

Mice were maintained on a C57BL/6 background. *Nkx3.2*-Cre (Nkx3-2^tm1(cre)Wez^) mice (45) were a generous gift from Warren Zimmer (Texas A&M University, College Station, TX), and *R26*-EYFP (Gt(ROSA)26Sor^tm1(EYFP)Cos^) and *Il33* ^flox/flox^-eGFP (Il33^tm1.1Bryc^) (46) mice were obtained from the Jackson Laboratory (**Table S1**). Wild-type mice were purchased from Envigo Ltd. (Jerusalem, Israel). Animals were housed under specific pathogen-free conditions. All experimental protocols were approved by the Tel Aviv University Institutional Animal Care and Use Committee (IACUC). The study was carried out in compliance with the ARRIVE guidelines.

### 2.2 Treatments

For glucose tolerance tests, mice were fasted overnight before the i.p. injection of dextrose (2 mg/gr). For insulin tolerance tests, mice were fasted 6 hours before i.p. insulin administration (0.5 U/kg). Tail vein blood glucose levels were measured at indicated time points using glucose meters (Bayer). When indicated, mice were i.p. injected every other day with either 500 ng murine rIL-33 (BioLegend; Cat #580502) or PBS to obtain a total of three doses. Mice were analyzed 24 hours after the last injection.

### 2.3 Islet isolation

For islet isolation, collagenase P solution (0.8 mg/ml; Roche) was injected through the common bile duct into the pancreas of the euthanized mouse, followed by 11 minutes of incubation and a density gradient (Histopaque-1119, Sigma) separation. Islets were collected from the gradient interface and hand-picked.

### 2.4 Flow cytometry

To obtain single cell suspension from whole pancreas, dissected tissue was incubated in HBSS supplemented with collagenase P (0.4 mg/ml; Roche) and DNase I (0.1 ng/ml; Sigma-Aldrich). For analysis of islet cells, double-hand-picked islets were gently dispersed with Accutase (Sigma-Aldrich) solution for 5 minutes. For analysis of blood immune cells, tail vein blood was collected into EDTA-containing tubes, followed by a density gradient separation with Lymphoprep (StemCell Technologies). For analysis of splenic cells, dissected spleens were injected with collagenase D (1 mg/ml; Roche), sliced, and incubated at 37°C for 45 minutes. Then dissociated spleen was minced, strained, and washed. After supernatant aspiration, the pellet was resuspended in hypertonic ACK buffer to lyse erythrocytes for 2 minutes at room temperature.

For staining with surface markers, dispersed cells were incubated with Fc blocker (anti-CD16/CD32 antibody) for 15 minutes, followed by staining with the appropriate antibodies (**Table S2**) for 30 minutes on ice and incubation with DAPI (Sigma-Aldrich) to label dead cells.

Aldehyde dehydrogenase (ALDH) activity was determined using the ALDEFLUOR kit (StemCell Technologies) according to the manufacturer’s protocol. Briefly, dispersed cells from 200 islets were equally divided into two tubes, control and experimental. Control cells were incubated with ALDH inhibitor diethylaminobenzaldehyde (DEAB) for 15 minutes at 37°C to define the background fluorescence. Then, control and experimental tubes were incubated with activated fluorescent ALDH reagent for 35 minutes at 37°C. Following incubation and washes, cells were stained with appropriate antibodies.

Cells were analyzed by Cytoflex (Beckman Coulter) and analyzed with Kaluza software (Beckman Coulter).

### 2.5 Cell isolation

For pancreatic pericytes isolation, pancreatic cells were collected from adult YFP^Pericytes^ (*Nkx3.2*-Cre;*R26*-EYFP) mice when pericytes were identified based on the yellow fluorescence (32, 34). Pancreatic cells were isolated according to a previously published protocol (47). Briefly, dissected pancreatic tissues were incubated in HBSS supplemented with collagenase P (0.4 mg/ml; Roche) and DNase I (0.1 ng/ml; Sigma-Aldrich) for 30 minutes at 37°C with mild agitation. Ice-cold HBSS buffer was added to stop digestion. Tubes were centrifugated at 300 g for 5 minutes at 4°C, washed, and strained through a 70 µm cell strainer (Miltenyi Biotec) to collect single cells. Cells were resuspended in PBS (without calcium chloride and magnesium chloride) supplemented with 5% fetal bovine serum and 5 mM EDTA and re-strained through a 35 µm cell strainer (Corning). When indicated, single-cell suspension from wild-type or YFP^Pericytes^ mice were incubated with Fc blocker (anti-CD16/CD32 antibody; **Table S2**) for 30 minutes followed by 30 minutes incubation on ice with anti-PECAM1/CD31 or anti-CD45 antibodies (**Table S2**) to identify endothelial and immune cells, respectively. Prior to sorting, cells were incubated with DAPI (Sigma-Aldrich) to mark and exclude dead and late apoptotic cells. Pancreatic pericytes, endothelial, and immune cells were collected using FACSAria II or FACSAria III cell sorters (BD).

### 2.6 Glucose-stimulated insulin secretion

For *in vivo* analysis, dextrose (2 mg/gr) was i.p. injected after an overnight fast. At indicated time points, tail vein blood was collected, and serum was separated. For *ex vivo* analysis, freshly isolated islets were pre-incubated for 30 minutes in RPMI medium with low glucose (1.67 mM). For each experiment, ten size-matched islets were hand-picked under a stereotaxic microscope and transferred in 5 µl volume to the well of 96-well U-bottom untreated plate, containing 250 µl of either low (1.67 mM) or high (16.7 mM)-glucose media, followed by 1-hour incubation at 37°C degrees and 5% CO2. Alternatively, islets were pre-incubated for an hour in Krebs-Ringer buffer containing glucose (1.67 mM), followed by a 1-hour incubation with or without KCl (30 mM). Islet insulin content was extracted by 1.5% HCl in 70% ethanol solution, followed by lysis using TissueLyser II (Qiagen). Hormone levels were measured using a mouse ultrasensitive Insulin ELISA kit (Alpco).

### 2.7 Immunofluorescence

Dissected pancreatic tissues were fixed in 4% paraformaldehyde, followed by embedding in either O.C.T compound (Scigen) or paraffin. For paraffin-embedded tissues, heat-induced antigen retrieval in Citra buffer (BioGenex) was performed before the staining. Tissue sections were immunostained with indicated antibodies (**Tables S3, S4**) when control and transgenic tissues were stained in parallel. For TUNEL (Terminal deoxynucleotidyl transferase dUTP nick end labeling) assay, the Fluorescein *In Situ* Cell Death Detection Kit (Roche) was used according to the manufacturer’s protocol. Images were acquired using BZ-9000 BioRevo (Keyence) and SP8 confocal (Leica microsystems) microscopes.

### 2.8 Morphometric quantification

For analysis of functional vasculature, fluorescently labeled tomato lectin (1 mg/ml; Vector Laboratories) was intravenously injected. After 5 minutes, mice were euthanized, and their pancreas was extracted and fixed in 4% paraformaldehyde. Tissue cryosections were stained for insulin and imaged. For analysis of islet pericyte coverage, fixed pancreatic tissues of untreated mice were analyzed. Cryo-sections were immunostained for the pericytic marker Neuron-glial antigen 2 (NG2) and insulin and imaged. At least 50 islets per mouse, defined by their insulin expression, were analyzed to determine NG2- or lectin-positive areas per islet. For measurement of β-cell mass, fixed pancreatic tissues of untreated mice were analyzed. Paraffin-embedded sections were immunostained for insulin and counterstained with HCS CellMask Stain (Invitrogen) to mark the section area. Whole tissue sections, at least 100 µm apart, were automatically imaged. For each mouse, the acquired insulin-positive area was divided by the pancreatic area and multiplied by its weight. For analysis of the α-/β-cell ratio, glucagon- and insulin-positive areas were calculated for each islet when 50 islets per mouse were analyzed. For cell proliferation analysis, pancreatic tissues from postnatal day 5 pups were analyzed. Cryo-sections of at least 50 µm apart were stained for insulin and marker of proliferation Ki67 (**Table S3**). After imaging, at least 300 insulin-positive cells per pup were analyzed manually and the portion of the Ki67-positive cells out of the total number of insulin-positive cells was calculated. Images were acquired using Keyence BZ-9000 (Biorevo) and analyzed using ImageJ software (NIH).

### 2.9 Gene expression

Gene expression levels were detected with Taqman (Applied Biosystems) or SYBR green assays (Applied Biosystems) using indicated primers (**Table S5**) and normalized to GAPDH and Cyclophilin, respectively. Expression levels were determined using the StepOne cycler (Applied Biosystems). Published mouse pancreatic pericytes RNA sequencing (34) was deposited in ArrayExpress (https://www.ebi.ac.uk/arrayexpress/experiments/E-MTAB-5325/). Published islet genomic data (48) was accessed in http://www.gaultonlab.org/pages/Islet_expression_HPAP.html.

### 2.10 Statistical analysis

Analysis was carried out using an unpaired two-tailed Student’s t-test (Prism software v.9; GraphPad). P values of 0.05 or less were considered to be statistically significant. Statistically significant outliers were identified according to the Grubbs’ method and excluded from the analyses.

## 3 Results

### 3.1 Pericytes are the pancreatic source of IL-33

**Figure 1:**
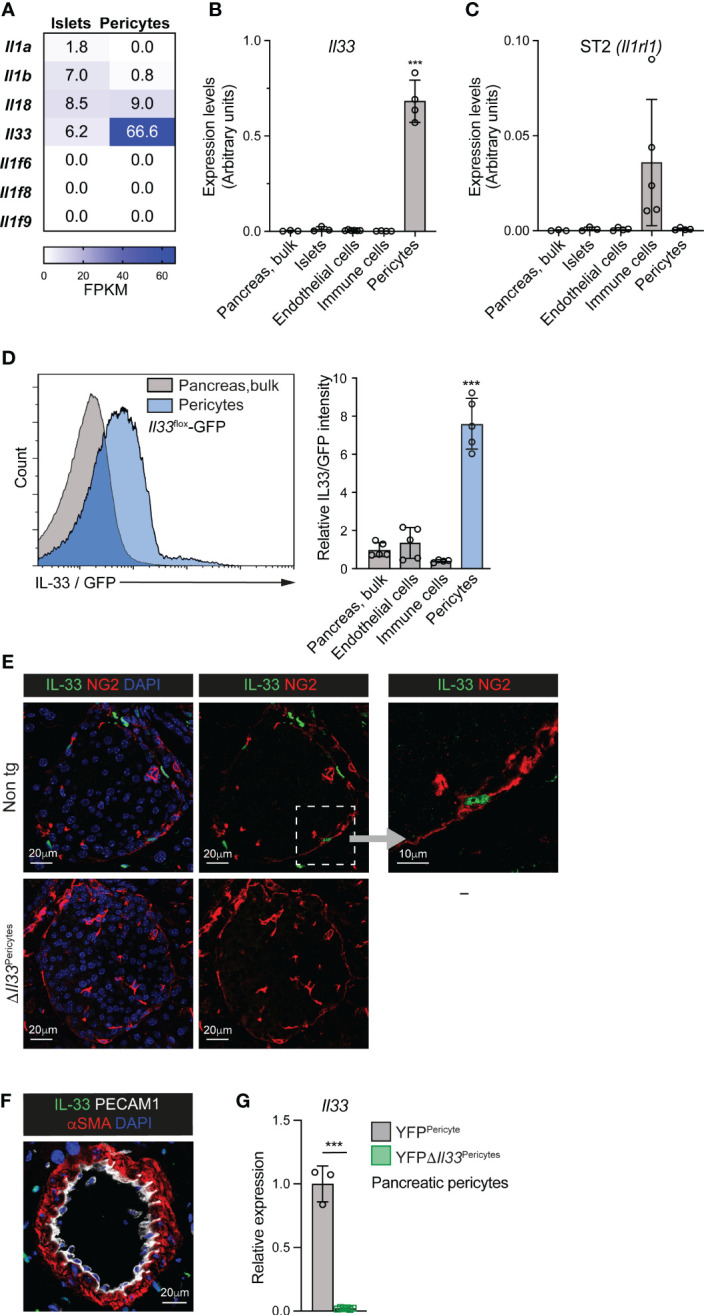
Pericytes are the primary source of IL-33 in the mouse pancreas and islet. **(A)** Analysis of IL-1 cytokine family ligands expression in isolated pancreatic pericytes and islets, employing a previously published RNAseq analysis (34). Heat maps show mean expression levels (as fragments per kilobase of exon per million aligned fragments [FPKM]) of indicated genes. N = 3. **(B, C)** Bar diagram (mean ± SD) shows relative levels of *Il33* **(B)** and *Il1rl1* (encodes ST2; C’) transcripts in different pancreatic cell populations, analyzed by qPCR (normalized to GAPDH). RNA was extracted from bulk pancreatic tissue, isolated islets, pancreatic endothelial cells (FACS-purified based on their PECAM1 expression) and pancreatic immune cells (FACS-purified based on their CD45 expression) of adult wild-type mice, and pancreatic pericytes (FACS-purified from *Nkx3.2*-Cre;*R26*-EYFP mice based on their yellow fluorescent labeling). N = 3-5. **(D)** Flow cytometry analysis of pancreatic tissue of *Il33* ^flox^-eGFP mice. Green fluorescence intensity was analyzed in bulk pancreatic cells, endothelial cells (defined as PECAM1^+^ cells), immune cells (defined as CD45^+^ cells), and pericytes (defined as PDGFRβ^+^ cells). *Left*, histogram showing representative fluorescence intensities of bulk pancreatic cells (gray) and pericytes (blue). *Right*, bar diagram shows the relative mean fluorescence intensity (MFI) when the average of bulk pancreatic cells was set to ‘1’. N = 4-5. **(E)** Immunofluorescence analysis of pancreatic tissue sections of non-transgenic (top) and *ΔIl33* ^Pericytes^ (bottom) mice. Sections were stained for IL-33 (green) and Neuron-glial antigen 2 (NG2; red) to label pericytes and counterstained with DAPI (blue). Representative islets (defined by morphology) are shown. *Right*, higher magnification of the area framed by the white box in the middle panel. **(F)** Immunofluorescence analysis of pancreatic tissue sections of non-transgenic mice. Sections were stained for αSMA (red) to label vSMCs, PECAM1 (white) to label endothelial cells, and IL-33 (green) and counterstained with DAPI (blue). Shown is a representative field. **(G)** Bar diagram (mean ± SD) showing lower *Il33* transcript levels in *Il33*-deficient pericytes. RNA was extracted from FACS-purified pancreatic pericytes from 3-month-old YFP*ΔIl33* ^Pericytes^ (*Nkx3.2*-Cre;*Il33* ^flox/flox^;*R26*-EYFP; green) and YFP^Pericytes^ (*Nkx3.2*-Cre;*R26*-EYFP; gray; the average was set to ‘1’) mice. Gene expression was analyzed by qPCR. N =3-5. ***p<0.005 (Student’s t-test). Each dot represents a single mouse.

IL-33 was shown to be produced by mesenchymal cells in the pancreas (7, 49). As pericytes are the predominant mesenchymal cell population in the islets (34, 50), we set to characterize their IL-33 expression. First, we analyzed the published transcriptome of pancreatic pericytes of naïve, healthy mice (34). As shown in **Figure 1A**, mouse pancreatic pericytes express IL-33 but no other members of the IL-1 family of cytokines. To define *IL33* expression in human islets, we employed a published islet cell transcriptome (48). Indeed, *IL33* is expressed by pericytes, identified by the pericytic markers *PDGFRB*, *ACTA2*, *ENG*, and *CD248* (30, 48)(**Figure S1**).

Next, we compared *Il33* transcript levels in various mouse pancreatic cell populations: bulk pancreatic tissue (contains mainly acinar cells), isolated islets (contains mainly endocrine cells), and purified pancreatic endothelial cells, immune cells, and pericytes. As shown in **Figure 1B**, our qPCR analysis revealed that *Il33* expression in pancreatic pericytes was two orders of magnitude higher than in other analyzed cell populations. Similarly, *IL33* expression in the human islets was restricted to the pericytic cell cluster (**Figure S1**) (48).

The expression of the IL-33 receptor ST2, encoded by *Il1rl1*, was previously shown to be restricted to ILC2s (7). Indeed, we detected its expression in immune cells but not in pericytes, endocrine, and endothelial cells of the mouse pancreas (**Figure 1C**).

To define pancreatic IL-33 expression further, we employed a transgenic mouse line in which GFP is expressed under this gene promoter, *Il33* ^flox^-eGFP (46). In agreement with our qPCR analysis, pericytes, but no other analyzed pancreatic cell populations, expressed IL-33/GFP (**Figure 1D**). Additionally, we performed immunofluorescence analysis to determine IL-33 protein expression. As shown in **Figure 1E**, we detected IL-33 protein in pericytes but not in other islet cells. Notably, although vascular smooth muscle cells (vSMCs) are closely related to pericytes, these cells did not express IL-33 (**Figure 1F**). Thus, pericytes constitute a predominant source of IL-33 in the islets.

### 3.2 Pericytic IL-33 is required for glucose regulation

To elucidate the role of IL-33 in pancreatic pericytes, we specifically deleted this gene in these cells. To this end, we crossed *Il33* ^flox^-eGFP mice, which allow Cre-dependent deletion of the *Il33* gene (46), with *Nkx3.2*-Cre mice (45) to generate *ΔIl33* ^Pericytes^ (*Nkx3.2-*Cre*;Il33* ^flox/flox^-eGFP) mice. As previously established, the expression of the *Nkx3.2*-Cre in the pancreas is restricted to the mural cell lineage (i.e., pericytes and vSMCs) and does not target epithelial or endothelial cells (32, 34, 50). Furthermore, *Nkx3.2*-Cre does not target immune cells in the pancreas, spleen, and blood (**Figure S2**). Immunofluorescence and qPCR analyses verified the loss of IL-33 expression in pancreatic pericytes of *ΔIl33* ^Pericytes^ mice (**Figures 1E, G**).

**Figure 2:**
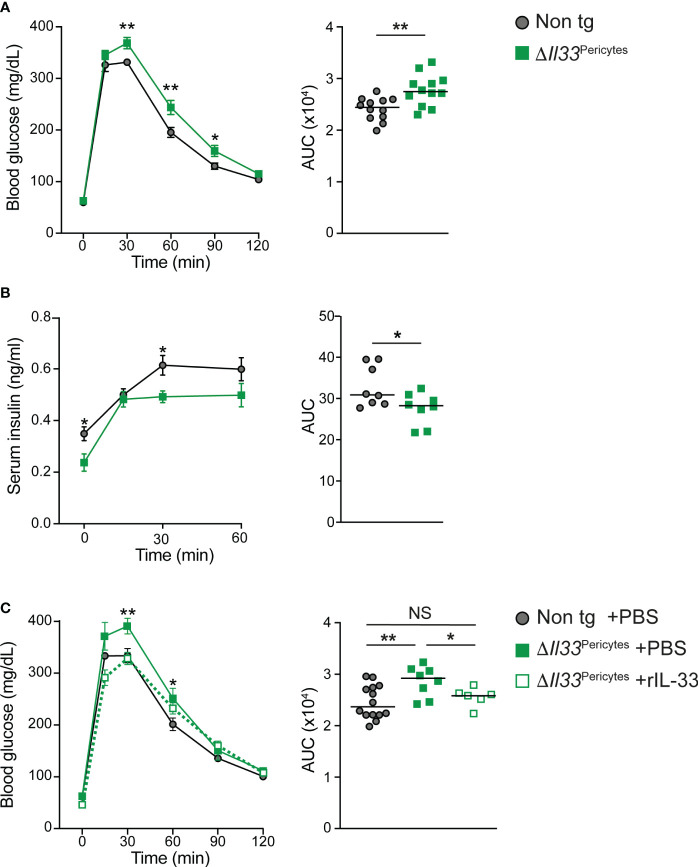
Impaired glucose response in mice lacking pericytic IL-33. 4-month-old *ΔIl33* ^Pericytes^ male mice (*Nkx3.2*-Cre;*Il33* ^flox/flox^; green), which lack *Il33* expression in their pancreatic pericytes, and non-transgenic littermates (Cre-negative; ‘Non tg’; gray) were analyzed. **(A)** i.p. glucose tolerance test (GTT). *Left*, mean (± SEM) blood glucose levels at indicated time points following glucose administration. *Right*, the area under the curve (AUC) of the response as shown on the left panel, when each dot represents a single mouse. N = 12. **(B)** *In vivo* GSIS. *Left*, mean (± SEM) serum insulin levels at indicated time points following glucose administration. *Right*, AUC of the response shown on the left panel, when each dot represents a single mouse. N = 8. **(C)** i.p. GTT after treatment of transgenic mice with exogenous IL-33. *ΔIl33* ^Pericytes^ mice were either i.p. injected with rIL-33 (500 ng/dose; dashed green line and empty squares) or PBS (solid green line and filled squares) every other day to obtain a total of three doses. In parallel, non-transgenic mice were i.p. injected with PBS. After an overnight fast, and 24 hours after the last treatment, mice were i.p. injected with dextrose (2 mg/g body weight), and tail vein blood glucose levels were measured at indicated times. *Left*, mean (± SEM) blood glucose levels at indicated time points following glucose administration. *Right*, AUC of the response as shown on the left panel, when each dot represents a single mouse. N = 6-14. *p<0.05; **p<0.01; NS, not significant, as compared with non-transgenic mice (Student’s t-test).

To determine if pericytic IL-33 is required for glucose regulation, we analyzed *ΔIl33* ^Pericytes^ and non-transgenic (Cre negative; *Il33* ^flox/flox^-eGFP) age- and sex-matched mice. Of note, it was previously shown that mice carrying the *Nkx3.2*-Cre allele alone display normal glucose response (34). *ΔIl33* ^Pericytes^ and non-transgenic control mice had comparable blood glucose levels (**Figure S3**). However, i.p. glucose tolerance test (GTT) analysis revealed a significantly impaired response to glucose challenge of *ΔIl33* ^Pericytes^ male mice (**Figure 2A**). We did not detect glucose intolerance in transgenic female mice (**Figure S3**). As *Il33* is expressed at similar levels in pancreatic pericytes of female and male mice (**Figure S3**), these differences likely reflect sex-dependent differences in glucose response (51). Correlating with their glucose intolerance, *ΔIl33* ^Pericytes^ male mice had significantly lower serum insulin levels after a glucose challenge than control mice (**Figures 2B**).

The *Nkx3.2*-Cre mouse line has non-pancreatic expression in the gastrointestinal mesenchyme and skeleton (45). We, therefore, analyzed for potential non-pancreatic phenotypes that may contribute to the glucose intolerance of *ΔIl33* ^Pericytes^ mice. Transgenic and control mice showed comparable body weight, indicating normal food uptake and digestion (**Figure S3**). Further, *ΔIl33* ^Pericytes^ male mice were insulin sensitive (**Figure S3**). Thus, our analysis demonstrated that pericytic IL-33 is required for proper glucose response by regulating insulin secretion.

### 3.3 Exogenous IL-33 rescues the glucose intolerance of Δ*Il33* ^Pericytes^ mice

The nuclear localization of IL-33 raised the possibility that this cytokine has a dual function and may also act cell autonomously in a receptor-independent manner (20, 21, 26). However, evidence accumulates that this cytokine acts primarily as a secreted ligand to induce signal transduction in ST2-expressing cells (22–24, 26). Notably, the loss of IL-33 affected neither islet pericytes abundance nor induced their activation (**Figure S4**). Further, IL-33 deficient islets had a comparable density of functional capillaries to control (**Figure S4**). To define if pericytic IL-33 acts as a secreted cytokine, we tested whether exogenous IL-33 rescues glucose intolerance of *ΔIl33* ^Pericytes^ mice. To this end, we i.p. injected rIL-33 into transgenic mice. As shown in **Figure 2C**, rIL-33 administration improved the glucose response of *ΔIl33* ^Pericytes^ mice, making it comparable to that of non-transgenic control mice. To conclude, our analysis suggests that pericytic IL-33 acts paracrine to regulate glucose response.

### 3.4 Insufficient insulin secretion in the absence of pericytic IL-33

**Figure 3:**
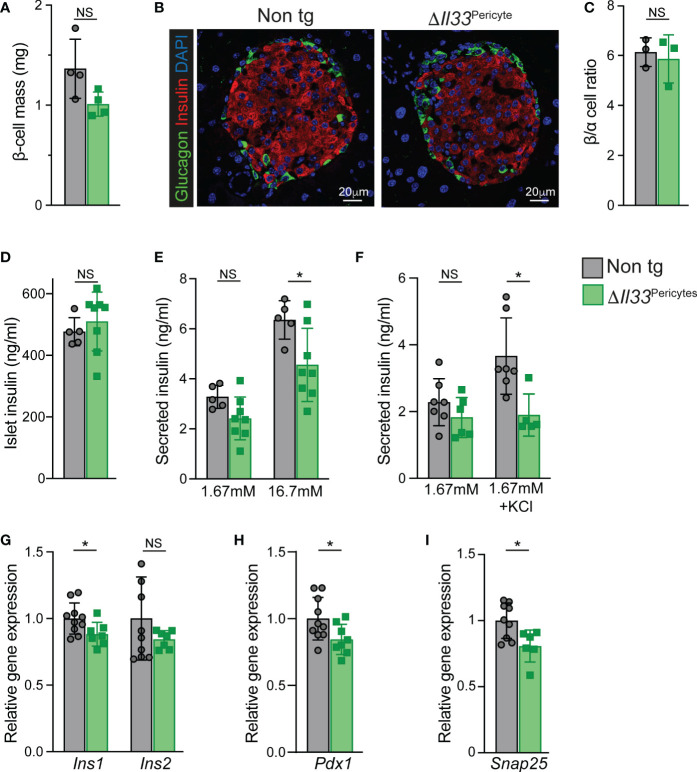
Impaired β-cell mass and islet insulin secretion in Δ*Il33* ^Pericytes^ mice. Islets and pancreatic tissues of *ΔIl33* ^Pericytes^ transgenic (green) and non-transgenic (‘Non tg’; gray). 4-month-old male mice were analyzed. **(A)** Bar diagram (mean ± SD) showing estimated β-cell mass. N = 4. **(B)** Pancreatic tissues of non-transgenic (left) and transgenic (right) mice were stained for insulin (red) and glucagon (green) and counterstained with DAPI (blue). Representative islets are shown. **(C)** Bar diagram (mean ± SD) showing the ratio between α- and β-cells (identified as glucagon and insulin-expressing cells, respectively) in adult pancreatic tissues of non-transgenic and transgenic mice. N = 3. **(D)** Bar diagram (mean ± SD) showing insulin content of size- and number- matched isolated islets. N = 5-8. **(E)** *Ex vivo* GSIS. Bar diagrams (mean ± SD) showing levels of insulin secreted from size-matched groups of ten isolated islets in response to 1.67 and 16.7mM glucose. N = 5-8. **(F)** Bar diagrams (mean ± SD) showing KCl-stimulated insulin secretion. Islets grouped into groups of ten islets were incubated with 1.67 mM glucose, either supplemented or not. N= 5-7. **(G–I)** Bar diagrams (mean ± SD) showing islet gene expression. Average levels in control islets were set to ‘1’. N = 6-10.*p<0.05; NS, not significant, as compared with non-transgenic control mice (Student’s *t-*test). Each dot represents a single mouse.

To define the underlying cause(s) of impaired insulin secretion of *ΔIl33* ^Pericytes^ mice, we analyzed their β-cells. The pancreatic mass of transgenic mice was comparable to the control when their β-cell mass was mildly, but non-significantly, lower (**Figures 3A**, **S5**). In agreement, we observed neither cell death nor expression of stress-related genes (i.e., *Chop*, *Atf4*) in *ΔIl33* ^Pericytes^ islets (**Figure S5**). Establishment of the β-cell mass relies on the proliferation of these cells in the neonatal period (52, 53). When analyzing β-cells of *ΔIl33* ^Pericytes^ and control pups (at postnatal day 5; p5), we observed comparable proliferation rates (**Figure S5**). Further, islet morphology and their β-/α-cell ratio were unaffected by the loss of pericytic IL-33 (**Figures 3B, C**). Thus, loss of pericytic IL-33 affected neither β-cell death nor their proliferation.

Next, we defined the islet insulin secretion *ex vivo* to determine blood flow-independent glucose-stimulated insulin secretion (GSIS). *ΔIl33* ^Pericytes^ islets displayed an impaired GSIS despite having comparable insulin content to non-transgenic islets (**Figures 3D, E**). Furthermore, transgenic islets secreted less insulin in response to KCl-mediated membrane depolarization (**Figure 3F**), pointing to abnormal islet insulin secretion in the absence of pericytic IL-33.

Gene expression analyses indicated slightly lower levels of *Ins1* and *Pdx1* in *ΔIl33* ^Pericytes^ islets but comparable levels of genes encoding other islet hormones or transcription factors (**Figures 3G, H**, **S6**). Further, we observed similar expression of genes encoding components of the GSIS machinery (**Figure S6**), but *Snap25*. This gene, which encodes a component of a SNARE complex that mediates the docking of insulin secretory granules to the plasma membrane (54), was slightly lower in *ΔIl33* ^Pericytes^ islets (**Figure 3I**). The moderately lower levels of *Ins1*, *Pdx1*, and *Snap25* in IL-33-deficient islets may thus contribute to their abrogated insulin exocytosis.

Overall, our analysis indicates a requirement for IL-33 for proper insulin secretion.

### 3.5 Deletion of pericytic IL-33 reduced islet T and dendritic cell numbers

Administration of rIL-33 affects islet immune composition (7). Thus, we aimed to define changes in immune cell numbers in *ΔIl33* ^Pericytes^ mice. First, we analyzed these mice for potential systemic effects on their immune cells by analyzing their spleen and blood. *ΔIl33* ^Pericytes^ and control mice had comparable portions of T cells in these tissues (**Figure S7**). Similarly, the levels of splenic DCs and blood monocytes (the precursors for DCs and some macrophages) were comparable in transgenic and control mice (**Figure S7**). Thus, we observe no systemic effect on analyzed immune cell populations in *ΔIl33* ^Pericytes^ mice.

**Figure 4:**
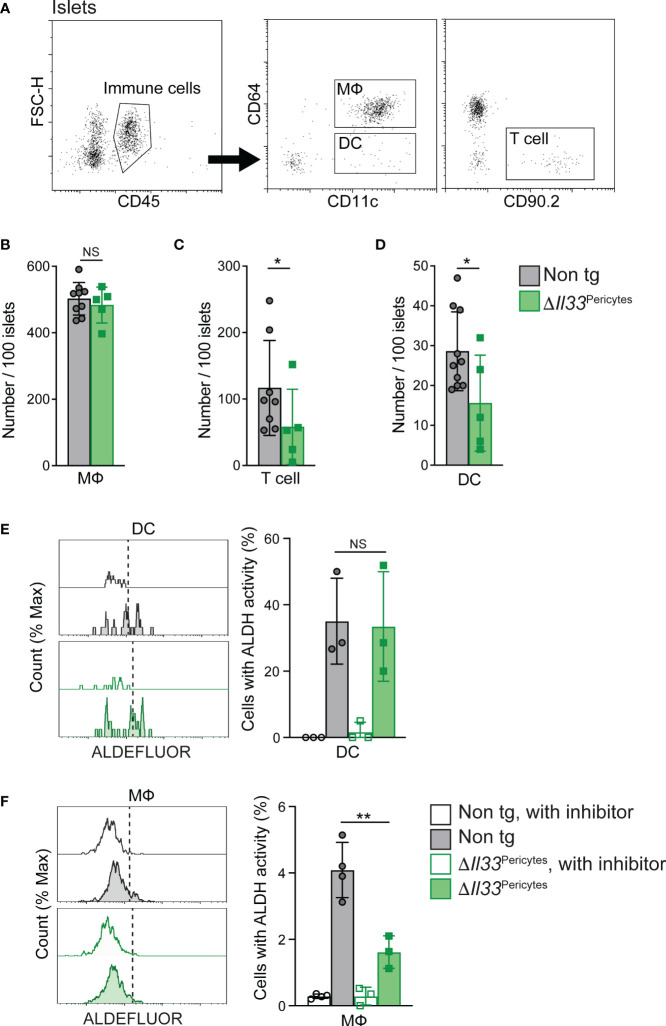
Loss of pericytic IL-33 affects islet immune cell number and functionIslets isolated from *ΔIl33* ^Pericytes^ (green) and non-transgenic (‘Non tg’; gray). 4-month-old male mice were analyzed by flow cytometry. **(A)** Representative dot plots indicating gate used to identify immune cells (Left panel, CD45^+^ cells), macrophages (MФ; Middle panel, CD45^+^CD11c^+^CD64^+^ cells), DCs (Middle panel, CD45^+^CD11c^+^CD64^-^ cells), and T cells (Right panel, CD45^+^CD90.2^+^ or CD45^+^CD3^+^ cells) out of dispersed islet cells. **(B–D)** Bar diagrams (mean ± SD) showing the total number of macrophages (MФ; B’), T cells (C’), and DCs (D’) in 100 isolated islets. N = 5-9. **(E, F)** Islet cells were analyzed with ALDEFLOUR to define the frequency of DC (E’) and macrophages (F’) with ALDH activity. Islets treated with the ALDH inhibitor diethylaminobenzaldehyde (DEAB; “with inhibitor”; empty bars and histograms) were used to define the background fluorescence for each sample, as indicated in the manufacturer’s protocol. *Left*, representative histograms. *Right*, bar diagrams (mean ± SD) showing the frequencies of cells with ALDH activity. N = 3-4. *p<0.05; ** p<0.01; NS, not significant, as compared with non-transgenic control mice (Student’s t-test). Each dot represents a single sample.

Next, to define a dependency of islet immune cells on pericytic IL-33, we analyzed *ΔIl33* ^Pericytes^ and non-transgenic islets (**Figure 4A**). We found no change in the number of islet macrophages or their ratio between cells with M1 and M2-like phenotypes (**Figures 4B**, **S7**). However, the loss of pericytic IL-33 caused a significant reduction in the number of T cells and DCs in the islets (**Figures 4C, D**). As these cell populations do not express ST2, they are unlikely to respond to IL-33 directly but depend on ILC2s activation (7). However, the scarcity of ILC2s in islets of C57BL/6 mice hindered their reliable analysis. To conclude, our analysis indicated that pericytic IL-33 is required to establish proper islet immune cell composition locally.

### 3.6 Impaired retinoic acid production capacity of islet macrophages in the absence of pericytic IL-33

rIL-33 was shown to indirectly promote islet RA synthesis to influence insulin secretion (7). RA is produced in two sequential oxidative steps: first, retinol is oxidized reversibly to retinaldehyde, and then retinaldehyde is oxidized irreversibly to RA; the latter is catalyzed by aldehyde dehydrogenases (ALDHs) (55). rIL-33 treatment enhanced ALDH activity by islet macrophages and DCs (7). To determine if pericytic IL-33 has a similar function, we measured the activity of ALDH in islet macrophages and DCs of control and *ΔIl33* ^Pericytes^ islets. As shown in **Figure 4E**, transgenic and control islets had a comparable portion of DCs with active ALDH. In contrast, the percentage of macrophages with active ALDH in *ΔIl33* ^Pericytes^ islets was a quarter of their portion in control islets (**Figure 4F**), implying that pericytic IL-33 is required for adequate RA production by these cells. Together with the overall decrease in DCs (**Figure 4D**), our analysis points to fewer RA-producing cells in *ΔIl33* ^Pericytes^ islets.

**Figure 5:**
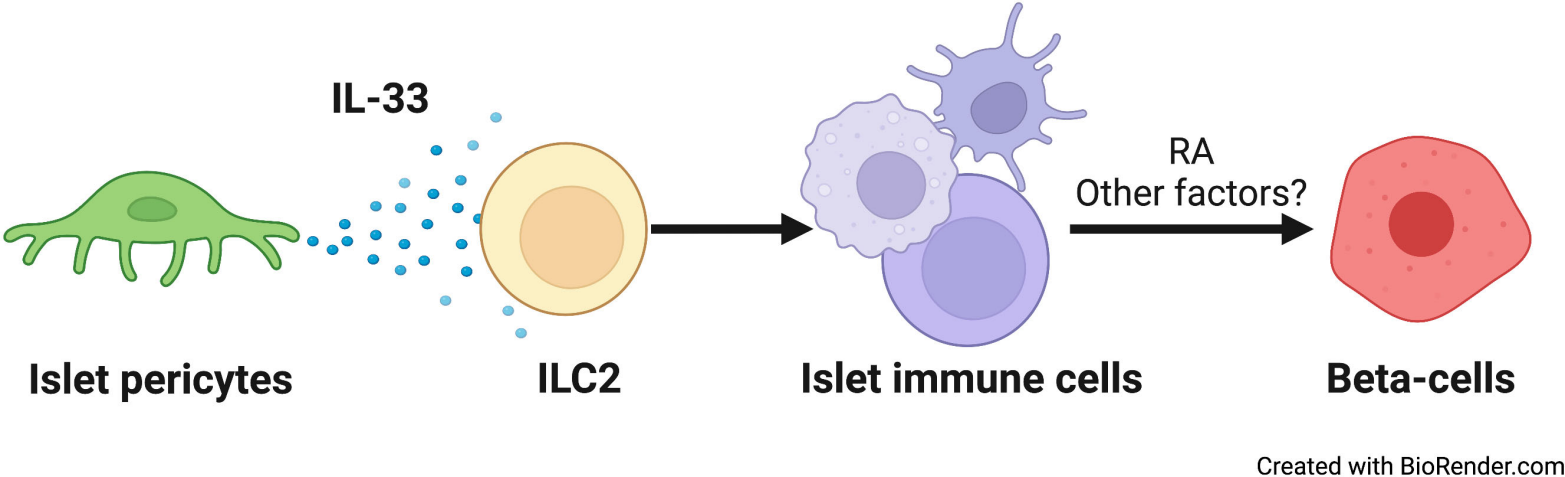
A schematic model of the relay effect of pericytic IL-33 on β-cells. From left to right: Islet pericytes (green) secrete IL-33. ILC2s (yellow) respond to this cytokine to affect other islet immune cells (yellow) (7). Islet T and dendritic cell numbers, as well as RA production by islet macrophages (7), are all sensitive to IL-33. IL-33-dependent RA secretion, and potentially other factors, promotes insulin secretion from β-cells.

## 4 Discussion

β-Cell function relies on the islet niche. Here, we provide evidence for pericyte-regulated islet inflammation and its role in glucose homeostasis. We showed that pericytes produce IL-33 and are the predominant cell type to do so. Pericyte-selective deletion of the *Il33* gene resulted in glucose intolerance due to impaired β-cell function. Pericytic IL-33 deficiency was further associated with fewer T cells and DCs in the islets. In addition, islet macrophages displayed lower ALDH activity in the absence of pericytic IL-33, implicating an impaired production of RA, which was associated with insulin secretion, by these cells. Thus, our study proposes that a cellular network comprised of pericytes and immune cells modulates β-cell function and insulin secretion (illustrated in **Figure 5**).

Administration of IL-33 was shown to improve glucose tolerance (7, 27). Dalmas et al. reported that systemic treatment with rIL-33 promotes insulin secretion (7). Despite some differences, our study indicated that administrated rIL-33 highly mimics the activity of the endogenous cytokine. Both exogenous and pericytic IL-33 promoted insulin secretion (7). Further, the number of islet DCs was affected by both rIL-33 and pericytic IL-33. However, while increasing IL-33 levels did not significantly affect islet T cell number (7), this cell population depends on the endogenous pericytic cytokine (**Figure 5**). Moreover, both endogenous and exogenous IL-33 affected RA production by islet macrophages, while RA production in DCs was only affected by increased cytokine levels (7). Thus, systemic administration of rIL-33 does not fully recapitulate its endogenous activity. Of note, systemic knockdown of *Il33* did not affect the glucose response of lean mice but only of diet-induced obese animals (7). These differences highlight the importance of pancreatic-specific production of IL-33 for glucose homeostasis.

The various cell populations that make the islet microenvironment interact to ensure proper insulin secretion. Neurons were shown to regulate islet blood flow and glucose uptake by activating pericytes (38, 56). Under stress, endothelial cells regulate the number and activity of macrophages, which produce factors promoting β-cell proliferation (1, 17, 18). The various islet immune cells cooperate to induce insulin secretion when ILC2s promote recruitment and RA production by islet macrophages and DCs (7). Our study introduces an additional player in this multi-cellular network: pericytes. As pericytes express other cytokines than IL-33, these cells may modulate islet inflammation and, thus β-cell functionality by regulating the number and activity of multiple immune cell populations. Further studies into the immunoregulatory activities of pancreatic pericytes are required to decipher their role in shaping islet inflammation and subsequent glucose homeostasis.

Cells of the islet niche, including pericytes and immune cells, support postnatal β-cell development, including functional maturation and proliferation (8, 32, 33, 36, 56). We observed no significant effect on β-cell mass and neonatal proliferation in the absence of pericytic IL-33. In contrast, this deletion impaired insulin secretion from β-cells, with little effect on their mature phenotype. While the expression levels of *Ins1* was slightly lower in islet lacking pericytic IL-33, this did not translate to reduced hormone levels. The expression of the transcription factor *Pdx1*, required for β-cell maturity, was reduced by 15% in *ΔIl33* ^Pericytes^ islets. While the lower *Pdx1* transcript levels may point to β-cell abnormalities, it is unlikely to significantly affect β-cell gene expression or functionality. Indeed, we did not observe changes in the expression levels of genes encoding proteins required for glucose sensing and insulin production. Thus, we suggest that pericytic IL-33 affects β-cell insulin secretion with little influence on these cells’ maturation and proliferation.

Throughout the body, pericytes were shown to regulate the immune system (39–44, 57, 58). In response to tissue damage, pericytes modulate the local immune response to promote repair. Brain pericytes modulate neuroinflammation by regulating immune cell recruitment and activation (39, 41, 42, 44, 57, 58). Kidney pericytes secrete cytokines in response to tissue damage (40, 43). Pericytes may also contribute to pathologies through immune cell activation. In the tumor stroma, pericytes produce IL-33, which recruits and activates tumor-associated macrophages, ultimately promoting metastasis (59, 60). Our study ascribes an immunoregulatory ability to pancreatic pericytes mediated by IL-33 production.

The clinical onset of type 2 diabetes (T2D) occurs when pancreatic β-cells fail to secrete sufficient insulin to maintain normoglycemia in the face of insulin resistance. A growing body of evidence has established T2D as an inflammatory disease associated with deleterious islet inflammation (1, 3, 17, 61–66). In particular, T2D is associated with an increased number of immune cells, predominantly macrophages, in the islets (11, 67). The shift from beneficial to harmful islet inflammation has been suggested to play a role in the progression of diabetes (61, 68). While obesity, a leading risk of T2D, increases IL-33 production in both human and mouse islets, it also interferes with the IL-33-ILC2 axis and its ability to promote insulin secretion (7). Islet pericytes are affected by genetic and metabolic risks of T2D (34, 38, 69, 70). Thus, by highlighting their immunoregulatory role, our study proposes that pericytes may contribute to the transformation of islet inflammation associated with diabetes. However, further research is needed to understand the interplay between pericyte function and T2D fully.

## Nomenclature

### Resource Identification Initiative

We take part in the Resource Identification Initiative and use the corresponding catalog number and RRID in our manuscript.

## Data availability statement

The datasets presented in this study can be found in online repositories. The names of the repository/repositories and accession number(s) can be found below: E-MTAB-5325 (EBI).

## Ethics statement

The animal study was reviewed and approved by Tel Aviv University Institutional Animal Care and Use Committee.

## Author contributions

GB, AS, LS, AE, TW, and MT conducted experiments and acquired and analyzed data. LL designed and supervised research and wrote the manuscript. All authors contributed to the article and approved the submitted version.
