# Supplementary material for: Pericytes modulate islet immune cells and insulin secretion through Interleukin-33 production in mice
Source: Front Endocrinol (Lausanne). 2023 Mar 9;14:1142988. doi: 10.3389/fendo.2023.1142988 (PMC10034381; doi:10.3389/fendo.2023.1142988)
Supplement: Supplementary file 1 [file DataSheet_1.pdf]

Supplementary Material

Pericytes modulate islet immune cells and insulin secretion through Interleukin-33 production in mice

Guzel Burganova, Anat Schonblum, Lina Sakhneny, Alona Epshtein, Tomer Wald, Mika Tzaig, and Limor Landsman*

*** Correspondence:** Limor Landsman: limorl@tauex.tau.ac.il

1 Supplementary Figures and Tables

1.1 Supplementary Figures

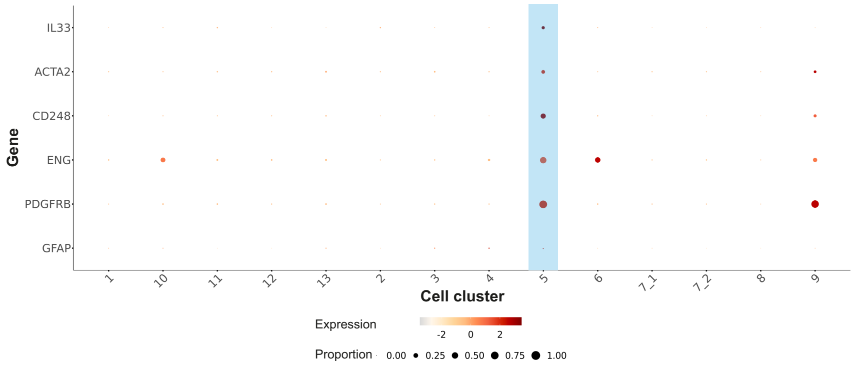

Supplementary Figure 1. ***IL33* is expressed by pericytes in human islets**

Analysis of *IL33* expression in isolated human islets, employing a published RNAseq analysis by the Human Pancreas Analysis Program (HPAP) (1) using the 'ShinyCell' application. Bubbleplot shows the gene expression patterns of *IL33*, the pericytic genes *ACTA2*, *PDGFRB*, *CD248*, and *ENG*, and the stellate cell gene *GFAP* grouped by categorical cell information (e.g., cell cluster). Note that *IL33* is expressed by cell cluster 5 (marked with a blue rectangle), which also expresses the analyzed pericytic genes.

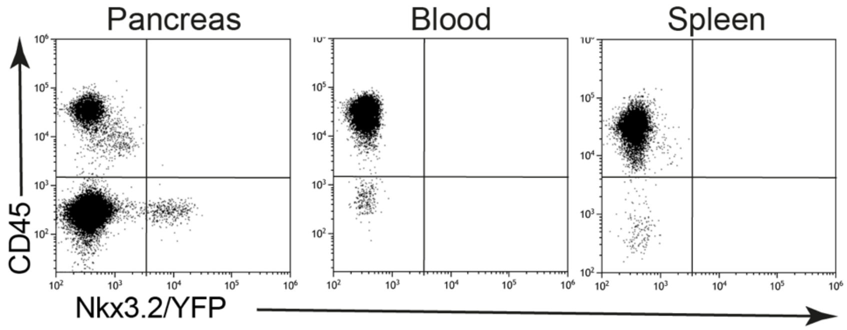

Supplementary Figure 2. Pancreatic, blood, and spleen immune cells are not targeted by the *Nkx3.2*-Cre line

Flow cytometry analysis of pancreatic, spleen, and blood cells from adult *Nkx3.2*-Cre;*R26*-YFP mice. Cells were stained for the immune cell marker CD45. Dot plots showing CD45 (y-axis) and yellow fluorescence ("Nkx3.2/YFP" x-axis) expression. Note no $CD45^+Nkx3.2/YFP^+$ cells (upper right quadrant) in the analyzed samples. Further, $CD45^-Nkx3.2/YFP^+$ cells (lower right quadrant) can be detected only in the pancreas.

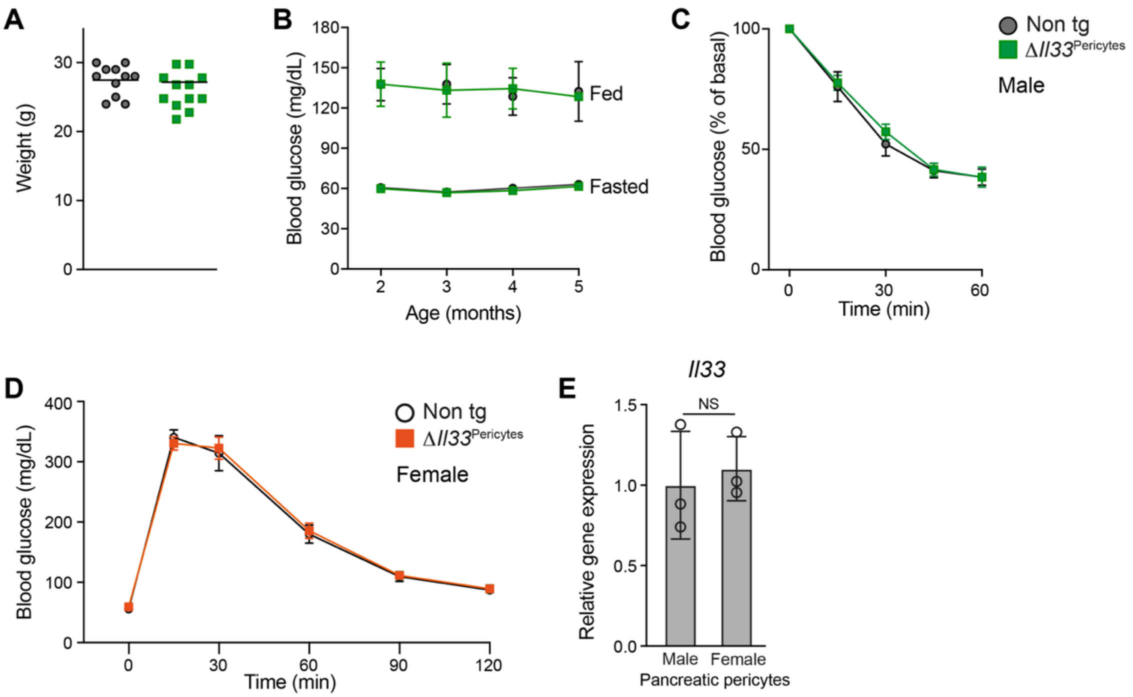

Supplementary Figure 3. Intact weight and insulin tolerance in pericytic IL-33 -deficient mice

Δ*Il33*^{Pericytes} mice (*Nkx3.2*-Cre;*Il33*$^{\mathrm{flox/flox}}$), which lack *Il33* expression in their pancreatic pericytes, and non-transgenic littermates (Cre-negative; 'Non tg') were analyzed.

(A) Body weight of 4-month-old Δ*Il33*^{Pericytes} (green) and non-transgenic (gray) male mice. Bar indicates the mean when each dot represents a single mouse.

(B) Blood glucose levels (mean ± SD) after an overnight fast ('Fasted') or upon *ad-lib* feeding ('Fed') of Δ*Il33*^{Pericytes} (green) and non-transgenic (gray) male mice at indicated ages. N ≥ 6.

(C) Intraperitoneal insulin tolerance test of 4-month-old Δ*Il33*^{Pericytes} (green) and non-transgenic (gray) male mice. Shown are mean (± SEM) blood glucose levels at indicated time points following insulin administration relative to baseline. N = 9-12.

(D) Intraperitoneal glucose tolerance test of 4-month-old Δ*Il33*^{Pericytes} (orange) and non-transgenic (gray) female mice. Shown mean (± SEM) blood glucose levels at indicated time points following glucose administration. N = 11-12.

(E) Bar diagram (mean ± SD) showing similar expression of *Il33* in pancreatic pericytes of female and male mice. RNA was extracted from FACS-purified pancreatic pericytes from YFP^{Pericytes} (*Nkx3.2*-Cre;*R26*-EYFP) male (the average was set to '1') and female mice. Gene expression was analyzed by qPCR. N = 3.

NS = not significant (Student's t-test).

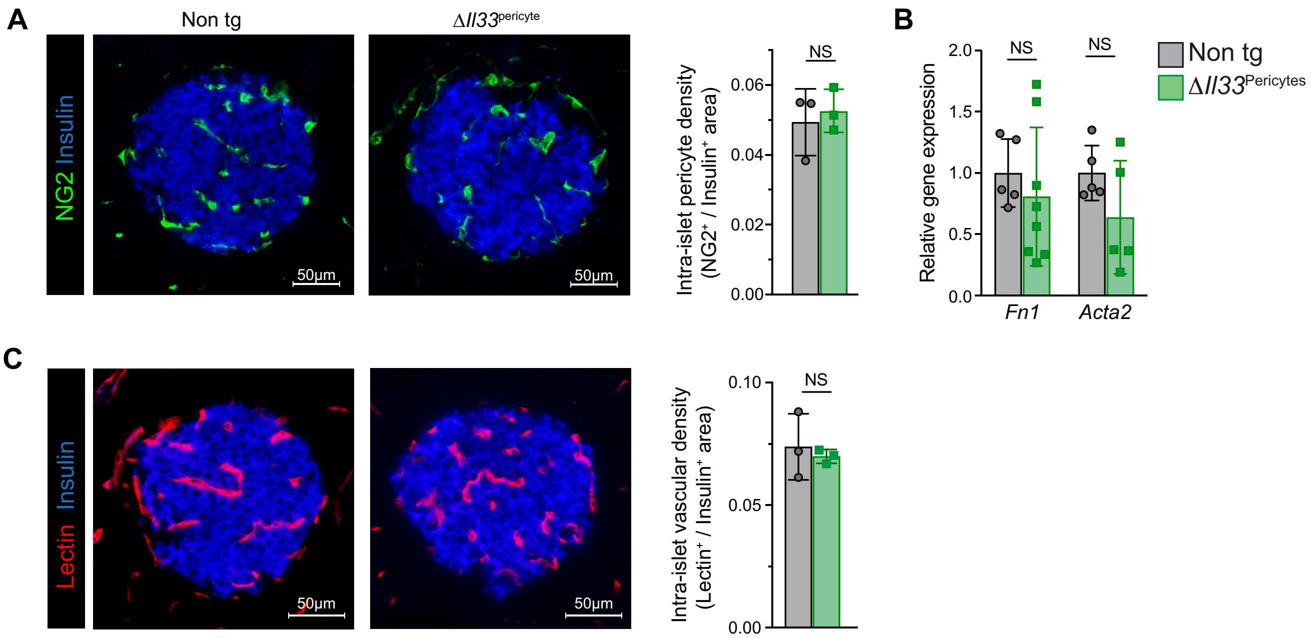

Supplementary Figure 4. No changes in islet vasculature upon *Il33* deletion

Δ*Il33*^{Pericytes} transgenic (green) and non-transgenic ('Non tg', Cre-negative; gray) 4-month-old male mice were analyzed.

(A) Analysis of pericytes intra-islet density. Pancreatic tissue cryosections were stained for NG2 (green) to label pericytes and insulin (blue) to label β-cells. *Left*, images of representative islets. *Right*, bar diagram (mean ± SD) showing a morphometric analysis of intra-islet pericyte density of ≥50 islets/mouse. The portion of NG2$^+$ area out of insulin$^+$ area was calculated per each islet. N = 3.

(B) Bar diagram (mean ± SD) showing unaffected expression of *Fn1* (encoding Fibronectin) and *Acta2* (encoding αSMA) in IL-33-deficient pericytes. RNA was extracted from FACS-purified pancreatic pericytes from YFPΔ*Il33*^{Pericytes} (*Nkx3.2*-Cre;*Il33*$^{\text{flox/flox}}$;*R26*-EYFP; green) and YFP^{Pericytes} (*Nkx3.2*-Cre;*R26*-EYFP; gray; the average was set to '1') mice. Gene expression was analyzed by qPCR. N = 5-8.

(C) Analysis of islet vasculature. Mice were intravenously injected with 1 mg/mL tomato lectin (red) to label functional blood vessels. After 5 minutes, the mice were euthanized, and their pancreas was harvested and fixed. Tissue cryosections were stained for insulin (blue). *Left*, images show representative islets. *Right*, Bar diagram (mean ± SD) showing quantification of intra-islet vascular density in ≥50 islets per mouse. The relative ratio of lectin$^+$ and insulin$^+$ area per each islet was calculated. N = 3.

NS = not significant (Student's t-test).

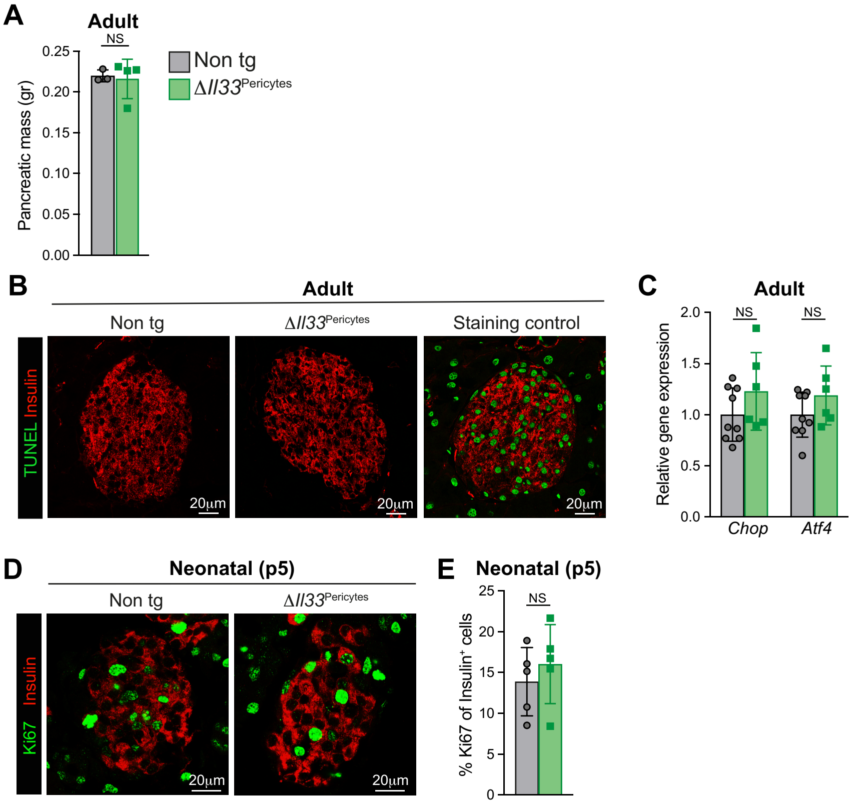

Supplementary Figure 5. β-cell survival and proliferation were unchanged in Δ*Il33*^{Pericytes} mice

Δ*Il33*^{Pericytes} transgenic (green) and non-transgenic ('Non tg', Cre-negative; gray) were analyzed.

(A) Bar diagrams (mean ± SD) showing the pancreatic mass of 4-month-old male mice. N = 3-4.

(B) Left and middle panels, pancreatic tissues of transgenic (middle panel) and non-transgenic (left panel) 4-month-old male mice were subjected to TUNEL assay (green) to identify dying cells and were stained for insulin (red) to identify β-cells. Right panel, non-transgenic pancreatic tissue pretreated with DNase to induce DNA breaks, which served as a positive control of the TUNEL assay ('staining control'). Shown are representative fields.

(C) Bar diagram (mean ± SD) showing gene expression analysis of isolated islets from 4-month-old male mice. RNA was extracted, and expression of the β-cell stress genes *Chop* and *Atf4* was analyzed by qPCR when average levels in non-transgenic islets were set to '1'. N = 6-9.

(D, E) β-Cell proliferation rate at postnatal day 5 (p5). D) Transgenic (right panel) and non-transgenic (left panel) pancreatic tissues stained with the proliferative marker Ki67 (green) and insulin (red). Shown are representative islets. E) Bar diagrams (mean ± SD) showing the portion of Ki67^{+}insulin^{+} cells out of insulin^{+} cells in pancreatic tissues stained as shown in D'. At least 300 insulin^{+} cells were analyzed for each mouse. N = 5.

NS = not significant (Student's t-test).

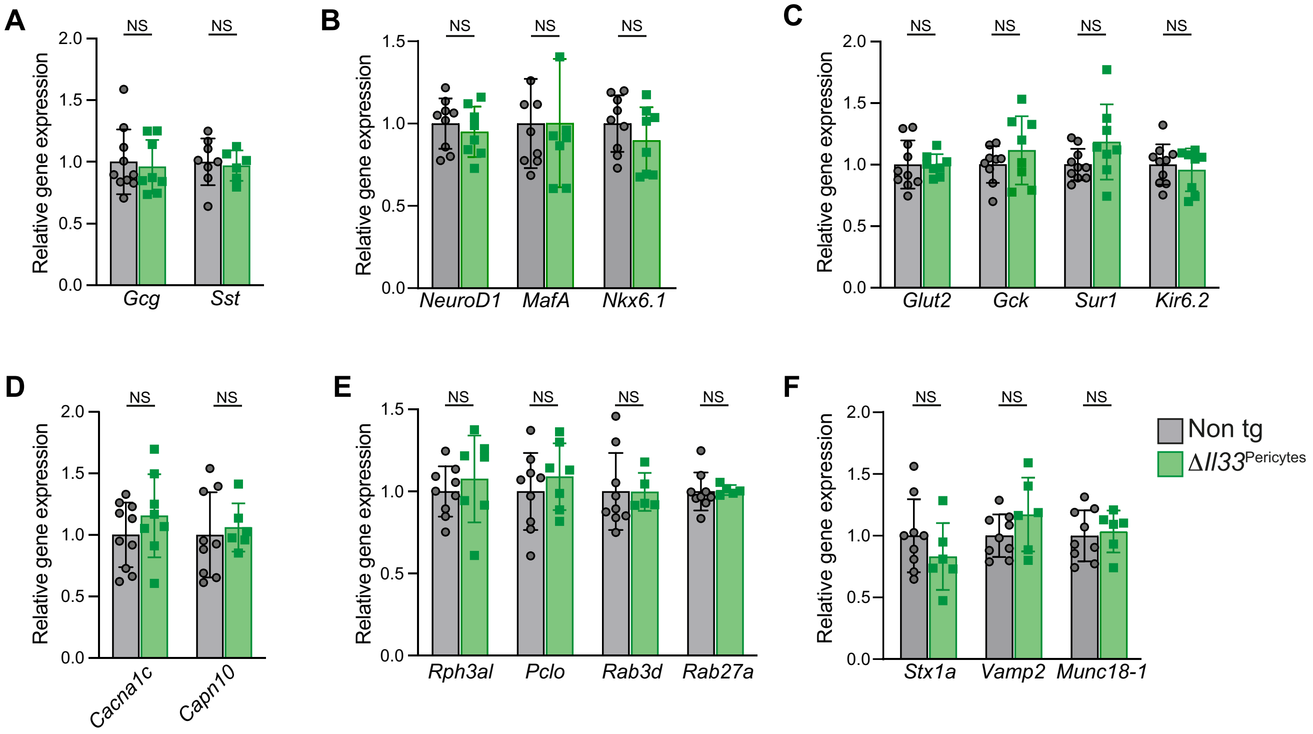

Supplementary Figure 6. Comparable islet gene expression in IL-33-deficient mice

Islets isolated from Δ*Il33*^{Pericytes} transgenic (green) and non-transgenic ('Non tg'; gray) 4-month-old male mice were analyzed by qPCR. Bar diagrams (mean ± SD) showing expression of indicated genes. Average levels in control islets were set to '1'. N = 6-10.

NS = not significant (Student's t-test). Each dot represents a single mouse.

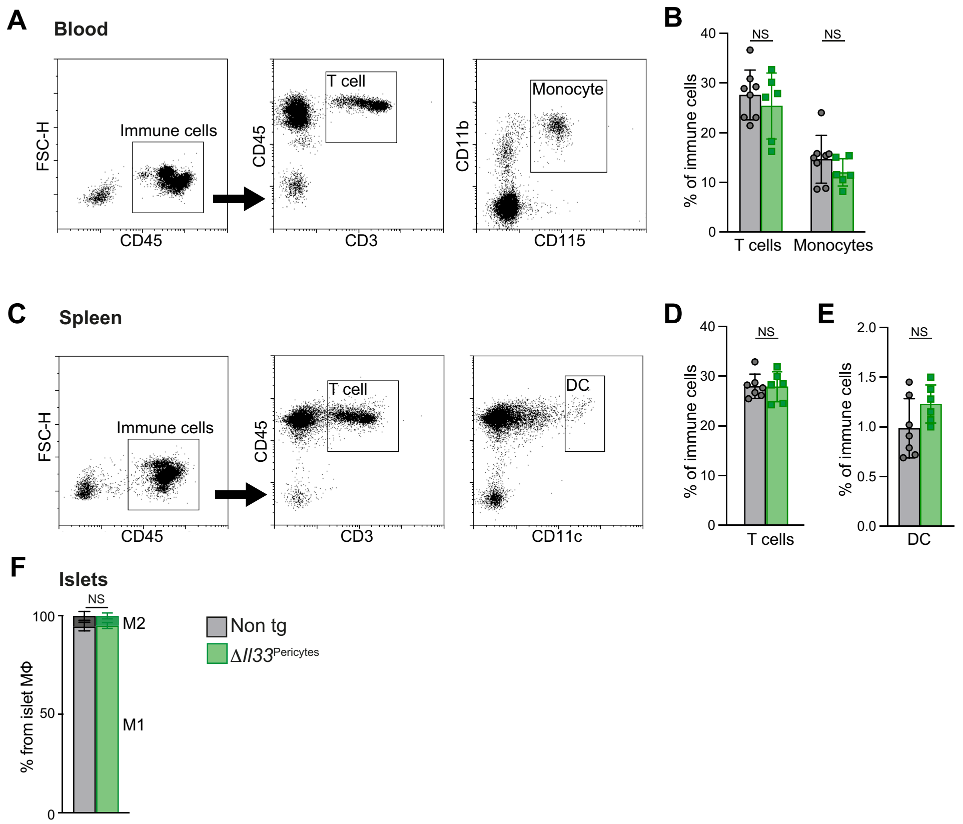

Supplementary Figure 7. Δ*Il33*$^{\mathbf{Pericytes}}$ mice display intact immune cell levels in the blood and spleen

Cells of Δ*Il33*^{Pericytes} transgenic (green) and non-transgenic ('Non tg', Cre-negative; gray) 4-month-old male mice were analyzed by flow cytometry.

(A) Representative dot plots indicating the gates used to identify blood immune cells ($CD45^+$ cells), T cells ($CD45^+CD3^+$ cells), and monocytes ($CD45^+CD115^+CD11b^+$ cells).

(B) Bar diagrams (mean ± SD) showing the portion of T cells ($CD3^+$ cells), and monocytes ($CD115^+$ $CD11b^+$ cells) out of total immune cells ($CD45^+$ cells). N = 6-8.

(C) Representative dot plots indicating gate used to identify spleen immune cells ($CD45^+$ cells), T cells ($CD45^+CD3^+$ cells), and DCs ($CD45^+CD11c^+$ cells).

(D, E) Bar diagrams (mean ± SD) showing the portion of T cells (D') and DCs (E') out of total immune cells. N = 6-7.

(F) Islet cells were analyzed. Bar diagrams (mean ± SD) showing the relative portion of cells with M1 ($CD206^-$ cells) and M2 ($CD206^+$ cells) -like phenotype out of total islet macrophages ($CD45^+CD64^+$ cells; MΦ). N = 4-5.

NS = not significant (Student's t-test). Each dot represents a single mouse.

1.2 Supplementary Tables

Supplementary Table 1. List of mouse strains

Mouse line	Name	Source	Identifier
Nkx3.2-Cre	Nkx3-2$^{tm1(cre)Wez}$	Warren Zimmer (Texas A&M University, College Station, TX)	PMID:19208343, RRID: MGI:3848005
R26-EYFP	B6.129X1-Gt(ROSA)26Sor$^{tm1(EYFP)Cos}$/J	The Jackson Laboratory	RRID: IMSR_JAX:006148
Il33^{flox}-eGFP	B6(129S4)-Il33$^{tm1.1Bryc}$/J	The Jackson Laboratory	RRID: IMSR_JAX:030619

Supplementary Table 2. List of antibodies for flow cytometry

Reagent	Source	Identifier
Armenian Hamster anti-CD11c, APC-conjugated (clone N418)	Thermo Fisher Scientific	Cat# 17-0114-81, RRID: AB_469345
Armenian Hamster anti-CD11c, PE-conjugated (clone N418)	BioLegend	Cat# 117307, RRID: AB_313776
Mouse anti-CD64 (FcγRI), FITC-conjugated (clone X54-5/7.1)	BioLegend	Cat# 139316, RRID: AB_2566556
Mouse anti-CD64 (FcγRI), PE-conjugated (clone X54-5/7.1)	BioLegend	Cat# 139304, RRID: AB_10612740
Rat anti-CD115 (CSF-1R), Alexa Fluor 488-conjugated (clone AFS98)	BioLegend	Cat# 135512, RRID: AB_11218983
Rat anti-CD11b, APC-conjugated (clone M1/70)	BioLegend	Cat# 101211, RRID: AB_312794
Rat anti-CD140b (PDGFR-β), biotin-conjugated (clone APB5)	BioLegend	Cat# 136010, RRID: AB_2236916
Rat anti-CD16/CD32 (clone 93)	Thermo Fisher Scientific	Cat# 14-0161-85, RRID: AB_467134
Rat anti-CD206 (MMR), PE-conjugated (clone C068C2)	BioLegend	Cat# 141705, RRID: AB_10896421
Rat anti-CD3, APC-conjugated (clone 17A2)	BioLegend	Cat# 100235, RRID: AB_2561455
Rat anti-CD31 (PECAM-1), PE-conjugated (clone 390)	BioLegend	Cat# 102407, RRID: AB_312902
Rat anti-CD45, APC-conjugated (clone 30-F11)	Thermo Fisher Scientific	Cat# 17-0451-82, RRID: AB_469392
Rat anti-CD45, FITC-conjugated (clone S18009F)	BioLegend	Cat# 157213, RRID:AB_2894427
Rat anti-CD45, PE-conjugated (clone 30-F11)	BioLegend	Cat# 103106, RRID:AB_312971
Rat anti-CD45, PerCP-conjugated (clone 30-F11)	BD Biosciences	Cat# 557235, RRID: AB_396609
Rat anti-CD90.2 (Thy-1.2), APC-conjugated (clone 53-2.1)	BioLegend	Cat# 140311, RRID: AB_10645337
Rat anti-TER-119/Erythroid cells, Pacific Blue-conjugated (clone TER-119)	BioLegend	Cat# 116232, RRID: AB_2251160
Streptavidin, APC-conjugated	Thermo Fisher Scientific	Cat# 17-4317-82

Supplementary Table 3. List of primary antibodies for immunofluorescence

Reagent	Source	Identifier	Used Dilution
Goat anti-IL-33 (polyclonal)	R&D Systems	Cat# AF3626, RRID:AB_884269	1:50
Guinea pig anti-insulin (polyclonal)	Agilent	Cat# IR002, RRID:AB_2800361	1:4
Rabbit anti-glucagon (polyclonal)	Millipore	Cat# AB932, RRID:AB_2107329	1:400
Rabbit anti-Ki67 (clone SP6)	Thermo Fisher Scientific	Cat# RM-9106-S0, RRID:AB_2341197	1:100
Rabbit anti-NG2 (polyclonal)	Millipore	Cat# AB5320, RRID:AB_91789	1:200
Rabbit anti-αSMA (polyclonal)	Abcam	Cat# ab5694, RRID:AB_2223021	1:200
Rat anti-PECAM1 (monoclonal)	BD Biosciences	Cat# 553370, RRID:AB_394816	1:200

Supplementary Table 4. List of secondary antibodies for immunofluorescence

Reagent	Source	Identifier	Used Dilution
Donkey Alexa Fluor 488-labeled anti-goat IgG (polyclonal)	Thermo Fisher Scientific	Cat# A-11055, RRID:AB_2534102	1:500
Donkey Alexa Fluor 555-labeled anti-rabbit IgG (polyclonal)	Thermo Fisher Scientific	Cat# A-31572, RRID:AB_162543	1:500
Donkey Alexa Fluor 555-labeled anti-rat IgG (polyclonal)	Abcam	Cat# ab150154, RRID:AB_2813834	1:500
Donkey Alexa Fluor 647-labeled anti-rabbit IgG (polyclonal)	Thermo Fisher Scientific	Cat# A-31573, RRID:AB_2536183	1:500
Goat Alexa Fluor 488-labeled anti-guinea pig IgG (polyclonal)	Thermo Fisher Scientific	Cat# A-11073, RRID:AB_2534117	1:500
Goat Alexa Fluor 488-labeled anti-rabbit IgG (polyclonal)	Thermo Fisher Scientific	Cat# A-11034, RRID:AB_2576217	1:500
Goat Alexa Fluor 555-labeled anti-guinea pig IgG (polyclonal)	Thermo Fisher Scientific	Cat# A-21435, RRID:AB_2535856	1:500

Supplementary Table 5. List of primers and probes for qPCR

Primers	Method	Sequence
Cacna1c	SYBR green	ATGAAAACACGAGGATGTACGTT<br>ACTGACGGTAGAGATGGTTGC
Cyclophilin	SYBR green	TGCCGCCAGTGCCATT<br>TCACAGAATTATTCCAGGATTC
Gck	SYBR green	GAGTGCTCAGGATGTTAAGGATCTG<br>GCTTTTGAGACCCGTTTTGTG
Ins1	SYBR green	GGGTCGAGGTGGGCC<br>CTGCTGGCCTCGCTTGC
Ins2	SYBR green	GGCTGCGTAGTGGTGGGTCTA<br>CCTGCTCGCCCTGCTCTT
Kcnj11(Kir6.2)	SYBR green	GGACCTCCGAAAGAGCATGA<br>GCGCACCACCTGCATGT
MafA	SYBR green	GCTGGTATCCATGTCCGTGC<br>TGTTTCAGTCGGATGACCTCC
Munc18-1	SYBR green	CTCACCCCACAGAAACTGCT<br>CAGCAGAGTAACCGAGGTGG
NeuroD1	SYBR green	ATGACCAAATCATACAGCGAGAG<br>TCTGCCTCGTGTTCCTCGT
Nkx6-1	SYBR green	TCAGGTCAAGGTCTGGTTCCA<br>CGGTCTCCGAGTCCTGCTT
Pclo	SYBR green	TACTCGGACCCATTTGTGAA<br>TACTGTTTGATTCCACTCGGGATT
Pdx1	SYBR green	CCCCAGTTTACAAGCTCGCT<br>CTCGGTTCCATTCGGGAAAGG
Rab27a	SYBR green	GGAGGCCCGGGAACTTG<br>TCTCAATCGCGTGGCTTATG
Rab3d	SYBR green	GGTCTACCGACATGACAAGAGGAT<br>GCGATAGTAGGCCGTGGTGAT
Rph3al	SYBR green	GCAGTGGAAATGATCAGTGG<br>TCAGGCACTGGCTCCTCCTC
Slc2a2 (Glut2)	SYBR green	TCAGAAGACAAGATCACCGGA<br>GCTGGTGTGACTGTAAGTGGG
Snap25	SYBR green	CAACTGGAACGCATTGAGGAA<br>GGCCACTACTCCATCCTGATTAT
Stx1a	SYBR green	CATGGACTCCAGCATCTCGAA<br>TCCATGAACATGTCGTGCAGC
Vamp2	SYBR green	GGGAGTCTGGACTTTTGGGG<br>GGGAAACGGGGTAAGGGAAG
Abcc8 (Sur1)	Taqman	Mm00803450_m1
Acta2	Taqman	Mm01546133_m1
Atf4	Taqman	Mm00515324_m1
Capn10	Taqman	Mm00516167_m1
Ddit3 (Chop)	Taqman	Mm00492097_m1

Fn1	Taqman	Mm01256744_m1
GAPDH	Taqman	TGCACCACCAACTGCTTAG<br>GGATGCAGGGATGATGTTC<br>Probe: CAGAAGACTGTGGATGGCCCCTC
Gcg	Taqman	Mm01269055_m1
Il1rl1	Taqman	Mm00516117_m1
Il33	Taqman	Mm00505403_m1
Sst	Taqman	Mm00436671_m1

References

1. Elgamal R, Kudtarkar P, Melton R, Mummey H, Benaglio P, Okino M-L, Gaulton KJ. An integrated map of cell type-specific gene expression in pancreatic islets. (2023) doi: 10.1101/2023.02.03.526994
